# Lipid kinase PIP5K1A regulates *let-7* microRNA biogenesis through interacting with nuclear export protein XPO5

**DOI:** 10.1093/nar/gkad709

**Published:** 2023-09-01

**Authors:** Chun Li, Bohyung Yoon, Giovanni Stefani, Frank J Slack

**Affiliations:** Harvard Medical School Initiative for RNA Medicine, Department of Pathology, Beth Israel Deaconess Medical Center, Harvard Medical School, Boston, MA 02215, USA; Harvard Medical School Initiative for RNA Medicine, Department of Pathology, Beth Israel Deaconess Medical Center, Harvard Medical School, Boston, MA 02215, USA; Harvard Medical School Initiative for RNA Medicine, Department of Pathology, Beth Israel Deaconess Medical Center, Harvard Medical School, Boston, MA 02215, USA; Harvard Medical School Initiative for RNA Medicine, Department of Pathology, Beth Israel Deaconess Medical Center, Harvard Medical School, Boston, MA 02215, USA

## Abstract

MicroRNAs (miRNAs) are small non-coding RNAs first discovered in *Caenorhabditis elegans*. The *let-7* miRNA is highly conserved in sequence, biogenesis and function from *C. elegans* to humans. During miRNA biogenesis, XPO5-mediated nuclear export of pre-miRNAs is a rate-limiting step and, therefore, might be critical for the quantitative control of miRNA levels, yet little is known about how this is regulated. Here we show a novel role for lipid kinase PPK-1/PIP5K1A (phosphatidylinositol-4-phosphate 5-kinase) in regulating miRNA levels. We found that *C. elegans* PPK-1 functions in the *lin-28*/*let-7* heterochronic pathway, which regulates the strict developmental timing of seam cells. In *C. elegans* and human cells, PPK-1/PIP5K1A regulates *let-7* miRNA levels. We investigated the mechanism further in human cells and show that PIP5K1A interacts with nuclear export protein XPO5 in the nucleus to regulate mature miRNA levels by blocking the binding of XPO5 to pre-*let-7* miRNA. Furthermore, we demonstrate that this role for PIP5K1A is kinase-independent. Our study uncovers the novel finding of a direct connection between PIP5K1A and miRNA biogenesis. Given that miRNAs are implicated in multiple diseases, including cancer, this new finding might lead to a novel therapeutic opportunity.

## Graphical Abstract

**Graphical Abstract:**
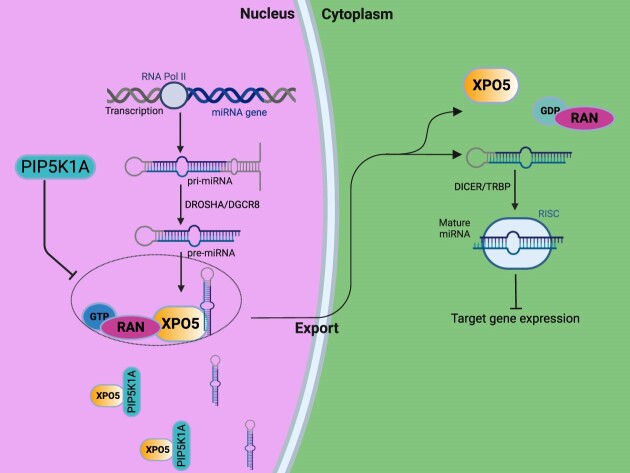

## INTRODUCTION

MicroRNAs (miRNAs) such as *lin-4* and *let-7* are small non-coding RNAs, which were first discovered through developmental studies in *Caenorhabditis elegans* (1,2). The *let-7* miRNA is highly conserved among species including humans (2,3). In the canonical miRNA biogenesis pathway, primary miRNAs (pri-miRNAs) are transcribed from their genetic locus by RNA polymerase II (Pol II) and processed into precursor miRNAs (pre-miRNAs) by the microprocessor complex (consisting of Drosha and DGCR8) and exported to the cytoplasm via an exportin 5 (XPO5)/Ran-GTP complex (4–6). In the cytoplasm, the pre-miRNA is further processed by the RNase III endonuclease Dicer into small RNAs of approximately 21 nucleotides and then loaded into Argonaute proteins in the RNA-induced silencing complex (RISC) (4,5). Finally, miRNAs interact with the 3′ untranslated region (3′ UTR) of target mRNAs to induce mRNA degradation and translational repression (4,5). During miRNA biogenesis, XPO5-mediated nuclear export of pre-miRNAs is a rate-limiting step for miRNA biogenesis (7,8) and, therefore, might be critical for the quantitative control of global miRNA levels.

The *C. elegans* heterochronic gene pathway regulates the timing of developmental events during post-embryonic development (9). This pathway consists of genes that encode RNA-binding proteins, such as LIN-28 and LIN-41, microRNAs, such as *lin-4* and *let-7* and transcription factors, such as *lin-14, hbl-1* and *lin-29* (1,2,10,11). *C. elegans* seam cells are lateral epidermal cells that divide during each larval stage with a stem-cell like pattern of cell fate and cell division (9,11). At each larval stage, one daughter of the cell division differentiates while the other daughter retains the ability to divide again. Around the beginning of the adult stage seam cells exit the cell cycle, fuse together and secrete a cuticular structure called alae (11). Mutations in heterochronic genes lead to temporal alterations to stage-specific patterns of cellular fate (11). For example, in precocious mutants, cells inappropriately express later cell fates during early stages, while in retarded mutants, cells reiterate earlier stage fates in place of later cell fates (12). These types of defects in developmental timing result in easily scorable phenotypes, for example by using the cell fusion marker *ajm-1::gfp* (apical junction marker), adult cell fate marker *col-19::gfp* (adult specific marker) and cell terminal differentiation markers such as alae formation. Two major components of the heterochronic gene pathway, LIN-28 and *let-7* miRNA, are evolutionarily conserved in animals where they have been shown to regulate each other's expression and have pivotal roles in pluripotency and differentiation (3,13,14). However, the mechanisms of action and downstream effectors of the *lin-28*/*let-7* pathway are poorly understood. Therefore, the identity of additional genes in the *lin-28*/*let-7* pathway in *C. elegans* will be important to better understand seam cell development and miRNA biogenesis.

Phosphatidylinositol 4-phosphate 5-kinase (PIP5K1A), is an enzyme that catalyzes phosphatidylinositol-4-phosphate (PI4P) to phosphatidylinositol-4,5-bisphosphate (PIP2) (15). Previous studies showed that PIP2 directly interacts with RNA polymerase II, and also binds with nuclear protein histone H1 to counteract the histone H1-mediated repression of basal transcription by RNA polymerase II (16,17). Larsson *et al.* showed human phosphatidylinositol 4-phosphate 5-kinase (PIP5K1A) has an important role in the PI3K/AKT signaling pathway that promotes prostate cancer cell proliferation and survival (18). *C. elegans* expresses only one PIP5K1A homologue, named PPK-1. Depletion of PPK-1 results in a defect in ovulation, reduced sterility and gonadal-sheath contractility, which is associated with Ins (1,4,5) P3 signalling (19).

In this study, we found that *C. elegans* PPK-1 regulates miRNA levels and functions in the *lin-28/let-7* heterochronic pathway. Interestingly, we also found PIP5K1A (the ortholog of PPK-1) regulates miRNA levels through interactions with nuclear export protein XPO5 in the nucleus, independent of its kinase activity. Furthermore, we demonstrated that PIP5K1A blocks the binding of XPO5 to pre-miRNA. Therefore, this study describes the novel finding of a direct connection between the lipid kinase PIP5K1A and miRNA biogenesis.

## MATERIALS AND METHODS

### *C. elegans* strains

The following *C. elegans* strains were used: N2 Bristol (wild-type)(CGC), lin-28(n719)(CGC), let-7(n2853) (2), syIs78[ajm-1::gfp + unc-119(+)] (20), maIs105[col-19::gfp] (21,22), wIs79 [ajm-1::gfp; scm-1::gfp] (20), lin-29(n546); maIs105[col-19::gfp] (23), let-7(n2853); maIs105[col-19::gfp] (24), lin-29(n546); wIs79[ajm-1::gfp; scm-1::gfp] (20) and let-7(n2853); wIs79[ajm-1::gfp; scm-1::gfp] (20). Transgenic animals were obtained as described. Pscm::mCherry::ppk-1 (25 ng/ul) was used in PPK-1 overexpression (OE) strain [Pscm::mCherry::ppk-1 + Pmyo-2::dsredm]. Chromosomal integration of extrachromosomal transgenes was performed using the UV integration method (25). The integrated animals were backcrossed with wild-type animals three times to clear background mutations. The Pscm::mCherry::ppk-1 plasmid was kindly provided by Dr Hitoshi Sawa. *C. elegans* strains were maintained at 20°C on nematode growth medium (NGM) plates overlaid with *Escherichia coli* OP50 strain, except for let-7(n2853) which was maintained at 15°C.

### RNA interference (RNAi)

RNAi experiments were performed at 20°C using *E. coli* HT115 bacteria expressing RNAi constructs from the Ahringer library (26). Transformed bacteria were overlaid on a nematode growth medium (NGM) plate containing 1mM IPTG and 50ug/ml Carbenicillin. Bacteria containing L4440 vector was used as a control.

### Microscopic analysis

L4 animals (P0) were placed on *ppk-1*(RNAi) plates and the F1 progeny was scored for seam cell number (*scm-1::gfp)*, seam cell fusion (*ajm-1;;gfp*), *col-19::gfp* expression and alae formation. *scm-1::gfp, ajm-1::gfp*, *col-19::gfp* expression and alae formation were observed using an upright Zeiss Axioplan microscope under 40x or 63x magnification. Animals were immobilized using 1mM levamisole on 2% agarose pads. Developmental stage was assessed by vulval and gonad development using DIC microscopy. Confocal images were acquired with a Carl Zeiss LSM 880 microscope using the Zen black software version SP2.3. The tile scan and z-stack images were acquired with a Plan-Apochromat 63x/ NA 1.4 objective lens with 10–16 images per condition. Cross-sections of a 3D volume reconstruction were generated using the Imaris image analysis software (Bitplane).

### Plasmids

pcDNA3.1-HA-XPO5 plasmid was generated by cloning the *xpo5* cDNA, amplified from HEK293 cells cDNA, into the pcDNA3.1-HA vector. pcDNA3.1-PIP5K1A-FLAG plasmid was generated by cloning the *pip5k1a* cDNA, amplified from HEK293 cells cDNA, into the pcDNA3.1-FLAG vector. The kinase-dead PIP5K1A mutant (*PIP5K1A*-D309N) was generated by replacing Asp-309 with Asn, which was performed by a Q5 Site-Directed Mutagenesis Kit (New England Biolabs). HA-KAP1 plasmid was kindly provided by Dr Dipanjan Chowdhury from Dana-Farber Cancer Institute/Harvard Medical School.

### RNA isolation and qRT–PCR

**Figure 1.**
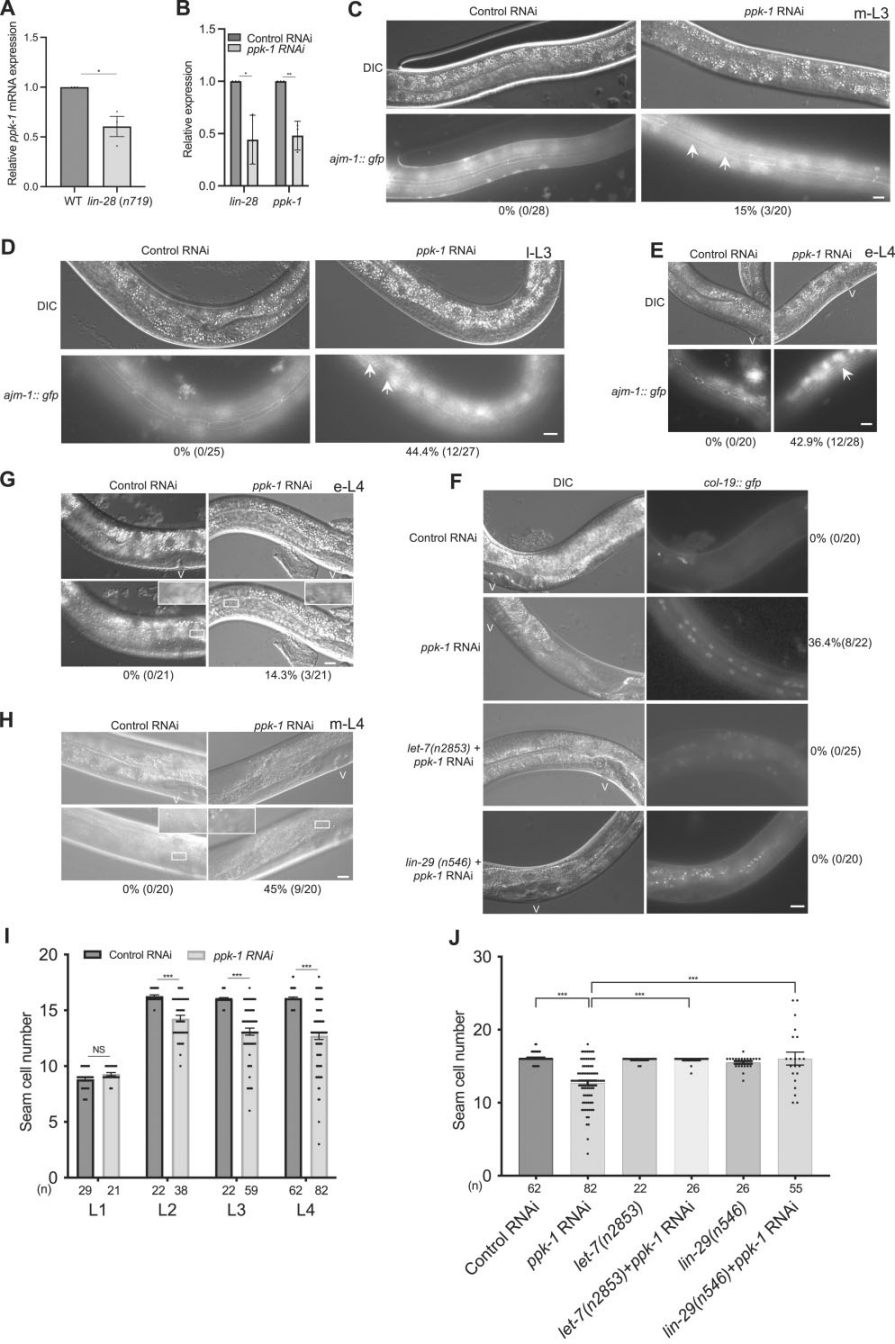
*ppk-1* is a new heterochronic gene. (**A**) qRT-PCR analysis of *ppk-1* mRNA levels in the wild type and *lin-28* (*n719*) mutant animals at the L2 stage. *pmp-3* mRNA was used as an endogenous control. (**B**) qRT-PCR analysis of *lin-28* mRNA and *ppk-1* mRNA levels from control RNAi and *ppk-1* (RNAi) animals at the peak time point of *lin-42* mRNA levels. *act-1* mRNA was used as an endogenous control. (**C–E**) *ajm-1::gfp* (Apical junction marker) expression in control RNAi and *ppk-1*(RNAi) animals at the mid-L3 (m-L3), late-L3 (l-L3) and early-L4 (e-L4) stages, respectively. The percentages of animals exhibiting any seam cell fusion and the number of animals examined are shown below the images. Arrows indicate the fusion of seam cells. V: Vulva. *n* ≥ 20, Scale bar: 10 μm. (**F**) *col-19::gfp* (Adult specific marker) expression in control RNAi, *ppk-1*(RNAi), *let-7(n2853)*; *ppk-1*(RNAi) and *lin-29(n546)*; *ppk-1*(RNAi) animals at the L4 stage. V: Vulva. *n* ≥ 20, Scale bar: 10 μm. The percentages of animals with *col-19::gfp* expression and number of animals examined are shown to the right of the images. (**G, H**) Precocious alae formation in control RNAi and *ppk-1*(RNAi) animals at early-L4 (e-L4) and mild-L4 (m-L4) stages, respectively. V: Vulva. *n* ≥ 20, Scale bar: 10 μm. The percentages of animals with alae formation and number of animals examined are shown below the images. (**I**) Seam cell number quantifications of control RNAi and *ppk-1*(RNAi) animals from the L1 stage to the L4 stage. *n* ≥ 20 (actual number shown below the bar). (**J**) Seam cell number quantifications from the indicated animals at the L4 stage. *n* ≥ 20 (actual number shown below the bar). All experiments were performed with at least three biological replicates. All data are represented as mean ± SEM. *P < 0.05, **P < 0.01, ***P < 0.001, ****P < 0.0001 and NS: not significant.

**Figure 2.**
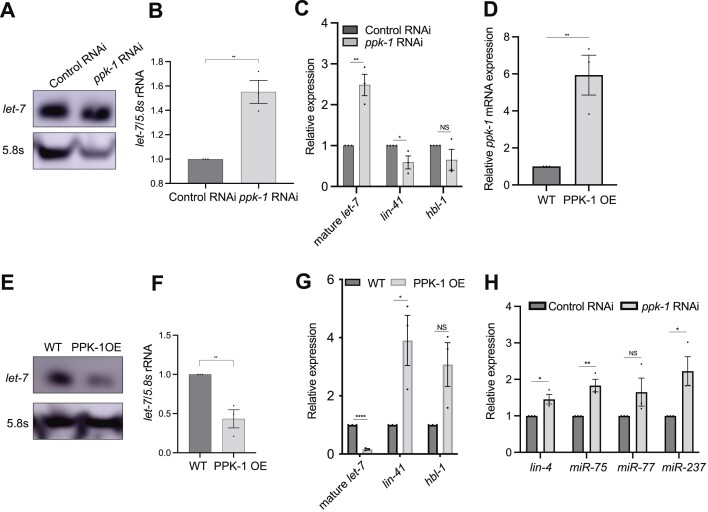
PPK-1 regulates miRNA expression and functions in *lin-28*/*let-7* heterochronic pathway. (**A, E**) Northern blot analyses of mature *let-7* expression from the indicated animals (at the peak time point of *lin-42* levels). 5.8S rRNA was used as a loading control. All experiments were performed with three biological replicates. (**B, F**) Quantitation of *let-7*/5.8S levels from northern blot analyses using ImageJ. (**C, G**) qRT-PCR analysis of mature *let-7, lin-41* and *hbl-1* levels from the indicated animals (at the peak time point of *lin-42* levels). miRNA levels were normalized to U18 snoRNA. *lin-41* and *hbl-1* mRNA levels were normalized to *act-1* mRNA. (**D**) qRT-PCR analysis of *ppk-1* mRNA levels in the wild type and PPK-1 OE animals. *act-1* mRNA was used as an endogenous control. **(H**) qRT-PCR analyses of mature *lin-4*, *miR-75, miR-77* and *miR-237* levels in control RNAi and *ppk-1*(RNAi) animals. U18 snoRNA was used as an endogenous control. All experiments were performed with at least three biological replicates. All data are represented as mean ± SEM. *P < 0.05, **P < 0.01, ****P < 0.0001 and NS: not significant.

Animals were bleached (5% 5 N NaOH, 10% bleach in M9 solution) starved in M9 (without *E. coli*) overnight to get synchronized L1s, which were put on plates for the experiments. qRT-PCR (Figures 1B and 2A–H) was performed at the peak time point of *lin-42* mRNA expression levels, as *lin-42* mRNA is dynamically expressed during development and peaks during the L4 stage (27).

Total RNA was collected in TRIzol (Invitrogen) and isolated using PureLink™ RNA Mini Kit (Invitrogen) or with Direct-zol Miniprep Plus spin columns (Zymo Research) according to the manufacturer's protocol, including the on-column DNase I treatment. cDNA synthesis was performed using High-Capacity RNA-to-cDNA™ Kit (Applied Biosystems) or High-Capacity cDNA Reverse Transcription Kit (Applied Biosystems). Total RNA concentration was measured using the NanoDrop Spectrophotometer (ND-1000 Spectrophotometer). mRNA expression levels were determined by quantitative RT-PCR using SYBR Green (Applied Biosystems) according to manufacturer protocols (Roche). MiRNA expression levels were determined by RT-qPCR using TaqMan MicroRNA Assays (Applied Biosystems). MiRNA levels in animals were normalized to U18 snoRNA, while mRNA or pri-miRNA levels were normalized with *pmp-3* or actin (*act-1)* mRNA levels and 5.8S rRNA served as control for normalization of pre-miRNA level. For mammalian RNA samples, miRNA or pre-miRNA and mRNA or pri-miRNA levels were normalized to U6 snRNA and *GAPDH* mRNA, respectively.

Nuclear and cytoplasmic RNAs were isolated following the protocol by Gagnon et al (28) and Choudhury et al (29). Briefly, RKO cells were lysed in ice-cold HLB (Hypotonic lysis buffer: 10 mM Tris, pH 7.5, 10 mM NaCl, 3 mM MgCl_2_, 0.3% NP-40 and 10% glycerol), supplemented with 100 U of Ribolock RNase inhibitor and incubated the mixture on ice for 10 min followed by centrifugation at 1000 g at 4°C. for 3 min. We carefully transferred the supernatant (cytoplasmic fraction) to a new tube and kept the pellet on ice. The nuclear pellet was washed with ice-cold HLB buffer three times and centrifuged at 300 g at 4°C. for 2 min. TRIzol was then added to both nuclear and cytoplasmic fractions and proceeded for RNA extraction. RT-qPCR was performed using TaqMan MicroRNA assays (Applied Biosystems), as described earlier. The purity of cytoplasmic and nuclear fractions was determined using *GAPDH* mRNA and *MALAT1*, respectively. Experiments were done in triplicate. For validation of the percent of pre-*let-7a-1*/pri-*let-7a-1* in the cytoplasmic-nuclear fractionation experiment, first we calculated the C/N ratio of pre-*let-7a-1* or pri-*let-7a-1* by using the 2^−ΔCt^[2^−(Cyt(ct)- Nuc(ct))^] method (ΔCt method). From this result, we calculated the C/N ratio of pre-*let-7a-1*/pri-*let-7a-1*, to get the percent of pre-*let-7a-1*/pri-*let-7a-1* in the cytoplasmic or nuclear fractions.

Primers (SYBR Green-based qPCR) for used in this study are:

- *act-1*-f: 5′-ACGCCAACACTGTTCTTTCC-3′
- *act-1*-r: 5′-GATGATCTTGATCTTCATGGTTGA-3′
- *pmp-3*-f: 5′-GTTCCCGTGTTCATCACTCAT-3′
- *pmp-3*-r: 5′-ACACCGTCGAGAAGCTGTAG-3′
- *ppk-1*-f: 5′-AAAGCTCGGACATCGACGAA-3′
- *ppk-1*-r: 5′-GAGACGCCAGACTTCCTATCG-3′
- *lin-28*-f: 5′-GCAAGGATTTCGGAGTCTTGATGAAGG-3′
- *lin-28*-r: 5′-GCAAACTTTCCACATCTGAAGCAACGTA-3′
- *lin-41*-f: 5′- GCTTCAGCAGTTGATGGCTAC-3′
- *lin-41*-r: 5′- CATCTCCACTTCCAACTGATCC-3′
- *hbl-1*-f: 5′-CTCGTCTAGTGACCCATTCT-3′
- *hbl-1-r:* 5′-ACGCCCGAACATTGATAAG-3 prime;
- Ce.5.8S rRNA-f: 5′-CTAGCTTCAGCGATGGATCGG-3′
- Ce.5.8S rRNA-r: 5′-CAACCCTGAACCAGACGTACC-3′
- *GAPDH*-f: 5′-TGCACCACCAACTGCTTAGC-3′
- *GAPDH*-r: 5′-GGCATGGACTGTGGTCATGAG-3′
- *PIP5K1A*-f: 5′-CTCCGGGCCGTCGTCTTCG-3′
- *PIP5K1A* -r: 5′-GCATAAGGCACCTCAGATGC-3′
- U6-f: 5′-CTCGCTTCGGCAGCACA-3′
- U6-r: 5′-AACGCTTCACGAATTTGCGT-3′
- *XPO5*-f: 5′-TGGCCACAGAGGTCACCC-3′
- *XPO5*-r: 5′-GGGCGCAGTGCCTCGTAT-3′

TaqMan probes (Thermo Fisher Scientific) are shown below.

U18 (Assay ID: 001764), cel-lin-4 (Assay ID: 000258), hsa-let-7a (Assay ID: 000377), cel-miR-75-3p (Assay ID: 000288), cel-miR-77-3p (Assay ID: 000230), cel-miR-237–5p (Assay ID: 462482_mat), pri-hsa-let-7a-1miRNA (Hs03302533_pri), pri-hsa-let-7b miRNA (Hs03302548_pri), Hsa-pre-let-7a-1 (Custom TaqMan Gene Expression assay, Assay ID: AP4729Y), GAPDH (Assay ID: Hs02786624_g1), β-actin (Assay ID: Hs01060665_g1), HMGA2 (Assay ID: Hs04397751_m1), LIN28A (Assay ID: Hs04189307_g1), MYC (Assay ID: Hs04189307_g1), MALAT1 (Assay ID: Hs00273907_s1), U6 snRNA (Assay ID: 001973), hsa-let-7b (Assay ID: 000378), has-let-7c (Assay ID: 000379), hsa-miR-125a-5p (Assay ID: 002198), hsa-miR-125b-5p (Assay ID: 000449), hsa-miR-886-5p (Assay ID: 002193), hsa-miR-122(Assay ID:002245).

### Northern blotting

Total RNA (20–40 ug) was extracted from nematodes or cells using TRIzol reagent (Invitrogen, USA) and northern blots performed using biotin-labeled probes following the protocol by Gagnon *et al.* (28). Briefly, total RNA samples were run on 15% PAGE–urea gels and transferred to Hybond-N+ positively charged nylon membranes (GE healthcare, USA) by electrophoresis. Next, the membranes were further UV-cross-linked and dried at 60°C for 1 h to improve binding. Before hybridization, the membranes were pre-hybridized for at least 1 h at 40°C in pre-hybridization buffer (#AM8677, Thermo Scientific). Next, hybridization buffer containing 50 pmol/ml biotin labeled single-stranded DNA oligonucleotide (See below) was added and the membranes were hybridized for overnight at 40°C with gentle shaking and subsequently rinsed with low stringency wash buffer (#AM8673, Thermo Scientific) 3 times and High stringency wash buffer 2 times (#AM8674, Thermo Scientific) at room temperature. The biotin-labeled probes were detected using a Chemiluminescent Nucleic Acid Detection Module Kit (#89880, Thermo Scientific) following the manufacturer's instructions. The bands were quantified using the ImageJ software.

- Hsa*-*let-7a: 5′-AACTATACAACCTACTACCTCA -3′/Bio/
- Ce.5.8S: 5′-GAACCAGACGTACCAACTGGAGGCCC -3′/Bio/
- Hsa.5.8S: 5′-TCCTGCAATTCACATTAATTCTCGCAG -3′/Bio/

### Immunoprecipitation

48h after transfection with the indicated plasmids, HEK293T cells were harvested and lysed with IP lysis buffer (Thermo Scientific™) supplemented with Protease and Phosphatase Inhibitor Cocktail (Thermo Scientific) and incubated on ice for 20 min. Human cells samples were centrifuged at 15 000 rpm at 4°C for 10 min. 5% of the supernatant was separated as input and the rest of the supernatant was incubated with anti-FLAG antibody (#F1804, Millipore sigma), anti-mCherry (#43590, CST) antibody, anti-HA magnetic beads (Thermo Scientific) and anti-PIP5K1A (pre-conjugated with protein A magnetic beads) overnight with constant rotation at 4°C. Later, beads were washed using wash buffer and the bound protein was eluted with 4× Laemmli Sample Buffer (Bio-Rad) by boiling for 5 min and further analyzed by western blot.

### Western blotting

Animals were lysed by boiling in SDS sample buffer while cells were harvested and lysed in RIPA lysis buffer (Thermo Scientific™) supplemented with protease inhibitor cocktail (Thermo Scientific). Total protein concentration was determined using Pierce BCA protein assay kit (Thermo Scientific). Proteins resolved by SDS page were transferred to PVDF membrane. Membranes were blocked with 5% milk protein in 1× TBST and incubated with primary antibodies overnight. Membranes were washed three times with 1× TBST and probed with a HRP-conjugated secondary antibody for 1 h followed by three additional washes. Specific antibody binding onto the membranes was detected using the SuperSignal™ West Pico PLUS Chemiluminescent Substrate (Thermo Fisher Scientific).

The following antibodies were used: V5-tag Polyclonal antibody (#14440-1-AP, Thermo scientific), Monoclonal ANTI-FLAG® M2 antibody (#F1804, Millipore sigma), DYKDDDDK Tag (D6W5B) Rabbit mAb (#14793, CST), GAPDH (14C10) Rabbit mAb (#2118, Cell signaling), Anti-HA antibody (#H3663, Millipore sigma), B-Actin antibody (#sc-47778, Santa Cruz Biotechnology), XPO5 Polyclonal Antibody (#PA5-93224, only recognizes the C-terminus of XPO5, URL: https://www.thermofisher.com/antibody/product/XPO5-Antibody-Polyclonal/PA5-93224), PIP5K1A Polyclonal Antibody (#15713-1-AP, Thermo scientific), Anti-rabbit IgG HRP-linked Antibody (#7074, Cell signaling), Anti-mouse IgG, HRP-linked Antibody (#7076, Cell signaling).

### Cell lines and transfection

HEK293T (obtained from the American Type Culture Collection (ATCC)) and RKO (Provided by Dr Kevin Haigis from Dana-Farber Cancer Institute of Harvard Medical School) cell lines were cultured in DMEM (high glucose) (Gibco, Cat #11995-065) supplemented with 10% FBS (Sigma-Aldrich) and 1% penicillin–streptomycin. Each cell line was maintained in a 5% CO_2_ atmosphere at 37°C.

To generate stable knockdown cell lines, *PIP5K1A* ShRNA (TRCN0000231477, Millipore Sigma) and control ShRNA (SHC016, Millipore Sigma) constructs were first transfected into HEK293T cells with the VSVG envelope vector and psPAX2 packaging vector using TransIT-Lenti reagent according to manufacturer's protocol (MirusBio). After 48 h, media was collected, centrifuged, and filtered through a 0.45 μm nitrocellulose filter. The virus was then added to RKO cells, and stably transduced cells were selected with puromycin and were maintained in 1.0 μg/ml puromycin media. To generate *PIP5K1A*-WT OE or *PIP5K1A*-D309N OE cells, pcDNA3.1-PIP5K1A-FLAG or pcDNA3.1-PIP5K1A- D309N-FLAG were transfected to RKO cells respectively, by using TransIT-X2 reagent (Mirus Bio, Madison, WI, USA) following the manufacturer's protocol. The plasmids and small interfering RNAs (siRNAs) were transfected to HEK293T or RKO cell lines using the TransIT-X2 reagent (Mirus Bio, Madison, WI, USA) following the manufacturer's protocol. Silencer select siRNAs were purchased from Santa Cruz Biotechnology: XPO5 siRNA (sc-45569) and Control siRNA (sc-37007).

### Immunofluorescent staining

Samples were fixed in 4% PFA for 10 min in phosphate-buffered saline (PBS) and washed three times for 5 min in PBS at room temperature, then incubated with 3% BSA in 0.3% Triton X-100 in PBS for 30min at room temperature. Samples were incubated with primary antibodies overnight at 4°C, and washed three times. Later they were incubated with secondary antibodies for 1 hour at room temperature, washed and mounted with ProLong™ Gold Antifade Mountant with DAPI ((#P36931, Thermo Fisher). Primary antibodies: PIP5K1A Polyclonal Antibody (# 15713-1-AP, Thermo Fisher scientific), XPO5 purified MaxPab mouse polyclonal antibody (B01P) (# H00057510-B01P, Abnova), Fluorescein conjugated Anti-PI (4,5) P2 IgM (#Z-G045, Echelon Biosciences), DYKDDDDK Tag (D6W5B) Rabbit mAb (Alexa Fluor® 594 Conjugate) (#20861, Cell signaling). Secondary antibodies: Goat anti-Rabbit IgG Secondary Antibody Alexa Fluor 488 (# A27034, Thermo Fisher scientific), Goat anti-Mouse IgG Secondary Antibody Alexa Fluor 568 (# A-11004, Thermo Fisher scientific). Confocal images were acquired with a Carl Zeiss LSM 880 upright confocal microscope, as described above. We define the C/N ratio of subcellular localization of XPO5 as the ratio of cytoplasmic to nuclear immunofluorescence intensity of XPO5, assessed by the mean fluorescence intensity in the cytoplasmic and nucleus volumes, which was performed by ImageJ software.

### RNA immunoprecipitation assay (RIP)

RKO control and PIP5K1A knockdown cells were transfected with plasmids expressing HA-XPO5. Forty-eight hours after transfection, the plates were rinsed twice in ice-cold PBS, cross-linked at 254 nm (120 mj/cm^2^), scraped and lysed in IP lysis buffer supplemented with Protease Inhibitor Cocktail and Ribolock RNase Inhibitor (40 U/μl; Thermo Fisher Scientific) on ice for 20 min and then centrifuged at 15 000 rpm speed at 4°C for 10 min. After centrifugation, 10% of the supernatant was separated as the input for qRT-PCR analysis. The rest of the supernatants were incubated with anti-HA magnetic beads or control magnetic beads with constant rotation at 4°C for overnight. The beads were washed 4 times with wash buffer. After washing, the beads were treated with proteinase K, and RNA was purified as described above. cDNA synthesis and PCR was performed as described above. After qRT-PCR analysis, the levels of pre-*let-7* and U6 in the IP samples were divided by the levels of them in the input calculated by a relative enrichment method, 2 ^(–(IP(ct)) – (input(ct)^. Fold enrichment of this ratio for HA IP normalized to the negative control IgG IP was determined.

### RNA pull-down assay

For GTP loading, 100uM GTP gamma S (#ab146662, Abcam) was incubated with Ran (#PRO-841, Prospec) protein and 20mM EDTA in the loading buffer (50 mM HEPES (pH 7.3), 200 mM NaCl, 5 mM MgCl_2_, 5 mM β-mercaptoethanol) at 30°C for 30 min. After loading, 60 mM MgCl_2_ was added and the reaction incubated on ice to stop the loading. RKO cells were transfected with HA-XPO5, lysed and XPO5 immunoprecipitated with anti-HA antibody and then eluted by IgG elution buffer (#21028, Thermo Fisher). Eluted HA-XPO5 protein was incubated with the above RAN-GTP, 3′ biotin labeled pre-*let-7a-1* (Purchased from GenScript) with or without of GST-PIP5K1A (#ab261922, Abcam) at 4°C for 4h, followed by incubation with Dynabeads MyOne Streptavidin C1 (#65001, Thermo Fisher) for another 1 h at 4°C. Later, the pull-downed proteins were washed using wash buffer and then subjected to western blotting by anti-HA and anti-GST antibodies (#2625, Cell Signaling). For detecting the RNA pull down efficiently, Streptavidin bead pull-downed pre-*let-7a-1* was run on 15% PAGE–urea gels and then stained by GreenGlo Dye (#CA3600, Fisher Scientific).

### Statistical analysis

All experiments were performed with at least three biological replicates. Statistical analysis was performed by Student's two- tailed *t* test in GraphPad Prism 9. Data points are presented as the mean ± SEM. *P*-values are: *P < 0.05, **P < 0.01, ***P < 0.001, ****P < 0.0001 and NS = not significant.

## RESULTS

### *ppk-1* is a heterochronic gene

*C. elegans* heterochronic genes in the *lin-28*/*let-7* pathway regulate the strict development timing of seam cells (2,11). We previously reported the use of CLIP-Seq to identify 2000 mRNAs interacting directly with the LIN-28 RNA binding protein (30), some of which were known heterochronic genes. To enrich for additional genes in the *lin-28*/*let-7* pathway, we determined the overlap among our set of LIN-28 CLIP hits (30), the set of 201 known *let-7* suppressors (31), 213 known *let-7* enhancers (32), and an analysis of *lin-28* dependent genes from our cursory RNA-seq data (Supplementary Table S1) from staged late larval stage 1 (L1) WT and *lin-28*(*n719*) mutant animals. We found 16 candidate genes that are at the intersection of these groups (Supplementary Figure S1). Of these genes, we focused on *ppk-1*, an ortholog of human PIP5K1A (phosphatidylinositol-4-phosphate 5-kinase type 1 alpha). While *ppk-1* was identified in the 213 known *let-7* enhancers (32) group, in that study, it did not show a strict positive relationship with *let-7*, as the vulval burst score was only 1.1 (32). PPK-1/PIP5K1A is an enzyme which produces phosphatidylinositol-4-phosphate (PI4P) to phosphatidylinositol-4,5-bisphosphate (PIP2), a lipid second messenger that regulates several cellular processes, such as signal transduction and vesicle trafficking (33).

Since PPK-1 was identified from LIN-28 CLIP-seq analysis, we tested if LIN-28 potentially regulates *ppk-1* mRNA levels. In *lin-28*(*n719*) mutants, *ppk-1* mRNA levels were decreased compared to wild type animals at the L2 stage (Figure 1A) (Supplementary Table S1). Surprisingly, *lin-28* mRNA levels also decreased in *ppk-1* RNAi compared to control RNAi animals (Figure 1B), suggesting that they function on each other in a feed forward loop.

To determine whether *ppk-1* is a heterochronic gene, we examined the well-characterized cell fusion marker *ajm-1::gfp* (Apical junction marker), alae formation (cell terminal differentiation marker) and adult cell fate marker *col-19::gfp* (adult specific marker) in temporally-staged *ppk-1*(RNAi) and control RNAi animals (the *ppk-1* null mutant exhibits lethality (34)). We confirmed efficient RNAi of *ppk-1* mRNA by qRT-PCR (Figure 1B). In control RNAi animals, seam cell fusion - as detected by *ajm-1::gfp* - occurs at the middle (mid) L4 stage, but is not observed at early stages (Figure 1C–E). However, 15% of *ppk-1*(RNAi) animals showed the *ajm-1::gfp* fusion phenotype at the mid-L3 stage, 44.4% of them at late-L3 stage and 42.9% of them at early-L4 stage (Figure 1C–E). Similarly, 36.4% of *ppk-1*(RNAi) animals showed precocious *col-19::gfp* expression at the L4 stage, but control RNAi animals did not (Figure 1F). Furthermore, we also found that *ppk-1*(RNAi) animals showed precocious alae formation at the early L4 stage (14.3%) and mid-L4 stage (45%) compared to the wild type which only showed this at the late L4 stage (Figure 1G, H). These results indicate that depletion of *ppk-1* causes a weak precocious heterochronic phenotype. Furthermore, as presented in Figure 1I, *ppk-1*(RNAi) animals showed a reduced seam cell number from L2 to L4 stages compared to control RNAi animals. Since these phenotypes are characteristic of precocious seam cell patterning, these results suggest that *ppk-1* is a new heterochronic gene.

### PPK-1 regulates miRNA levels and functions in the *lin-28*/*let-7* heterochronic pathway

One feature of precocious heterochronic mutants such as *lin-28* is altered expression of miRNAs involved in developmental timing, such as *let-7* (35). In order to determine whether PPK-1 regulates *let-7* miRNA expression, we performed qRT-PCR experiments over developmental time using *lin-42* as a reference gene, as its expression peaks once during each larval stage (27). *ppk-1*(RNAi) animals showed the peak time point of *lin-42* expression 3 hours earlier compared to control RNAi animals at 20°C (Supplementary Figure S2A-B).

At the peak time point of *lin-42* mRNA levels, we observed that *ppk-1*(RNAi) animals showed increased mature *let-7* levels compared to control RNAi animals by northern blotting and qRT-PCR analysis (Figure 2A–C). We also found that mature *let-7* levels increased in *ppk-1*(RNAi) animals compared to control RNAi animals at early stages such as m-L2 and e-L3 stage by a qRT-PCR assay, which was consistent with the precocious phenotypes of *ppk-1*(RNAi) animals (Supplementary Figure S3).

We examined whether over-expression (OE) of PPK-1 was sufficient to alter *let-7* expression, we created PPK-1 (OE) animals by integrating a *ppk-1* multi-copy array. When driven from its own promoter, the PPK-1 (OE) animals showed sterility and embryonic lethality phenotypes (not shown). However, as *ppk-1* was known to be expressed in seam cells from previous report (34), we created PPK-1 OE integrated animals driving *ppk-1* from a seam cell specific promoter. PPK-1 (OE) animals showed a 4 hour developmental delay compared to wild type animals (Supplementary Figure S2C, D), and showed increased *ppk-1* mRNA levels (Figure 2D). PPK-1 OE animals showed decreased mature *let-7* levels compared to wild type animals at the peak time point of *lin-42* levels, through northern blotting and qRT-PCR analysis (Figure 2E-G). These results suggest that PPK-1 negatively regulates the expression of mature *let-7*.

To test if *ppk-1* has a genetic interaction with *let-7* in the heterochronic pathway, we knocked down *ppk-1* in *let-7(n2853)* mutants. We found that the *let-7(n2853)* mutation suppresses the precious *col-19::gfp* phenotypes and decreased seam cell number of *ppk-1*(RNAi) animals (Figure 1F, J). *let-7* controls seam cell divisions by negatively regulating its targets *lin-41* and *hbl-1*. We found that the mRNA levels of *lin-41* significantly decreased or increased under depletion or overexpression of PPK-1 compared to control animals, respectively, however *hbl-1* mRNA levels were not significantly changed in both conditions (Figure 2C, G). *lin-29*, encodes a zinc-finger transcription factor that is required for hypodermal seam cell terminal differentiation, and functions downstream of *let-7* (36). We found that the *lin-29(n546)* mutation suppressed the precious phenotypes and decreased seam cell number of *ppk-1*(RNAi) animals, similar to the *let-7*(*n2853*) mutation (Figure 1F, J). These results indicate that PPK-1 functions upstream of *let-7*/*lin-41* and *lin-29*. We also tested the levels of other miRNAs, such as *lin-4*, *miR-75*, *miR-77* and *miR-237*, in *ppk-1*(RNAi) animals. Interestingly, most of those miRNA levels were also significantly increased in *ppk-1(*RNAi) animals (Figure 2H). Taken together, these results demonstrate that PPK-1 regulates miRNA levels and acts in the *lin-28*/*let-7* heterochronic pathway.

Since most of the *C. elegans* factors shown above have orthologs in mammalian cells, to further explore the detailed mechanism of how PPK-1 regulates mature *let-7* levels, we utilized mammalian cells going forward.

### PIP5K1A, the ortholog of *C*.*elegans* PPK-1 regulates miRNA expression

**Figure 3.**
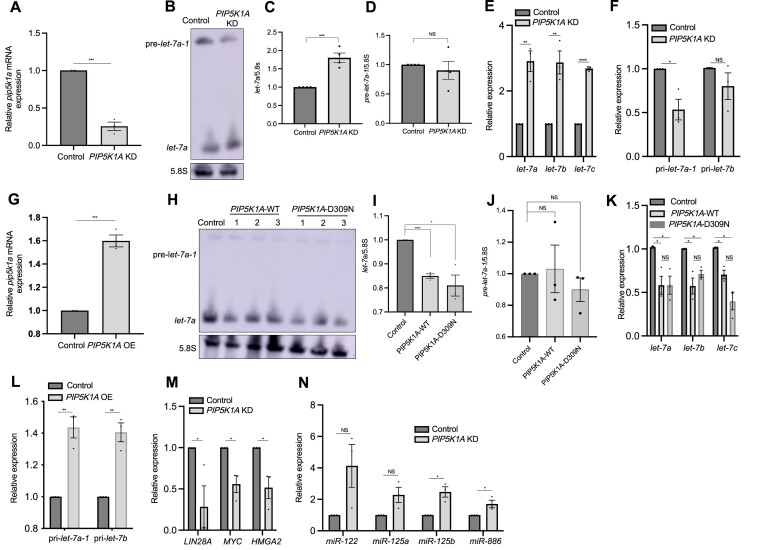
PIP5K1A regulates miRNA expression. (**A, G**) qRT-PCR analysis of *PIP5K1A* mRNA levels from indicated RKO cell lines. *GAPDH* mRNA was used as an endogenous control. (**B, H**) Northern blot analyses of mature *let-7a* and pre-*let-7a-1* expression from the indicated cells. 5.8S rRNA was used as a loading control. All experiments were performed with at least three biological replicates. (C, D, I, J) Quantitation of *let-7a*/5.8S (**C, I**) or pre-*let-7a-1*/5.8S (**D, J**) levels using Image J from the indicated cells. (**E, K**) qRT-PCR analysis of mature *let-7a, let-7b* and *let-7c* levels from indicated RKO cell lines. U6 snRNA was used as an endogenous control. (**F, L**) qRT-PCR analysis of pri-*let-7a-1* and pri-*let-7b* levels from indicated RKO cell lines. pri-miRNA levels were normalized to *GAPDH* mRNA. (**M**) qRT-PCR analysis of *LIN-28A*, *MYC* and *HMGA2* mRNA levels in control and *PIP5K1A* KD RKO cell lines. *GAPDH* mRNA was used as an endogenous control. (**N**) qRT-PCR analysis of mature *miR-122*, *miR-125a*, *miR-125b* and *miR-886* levels in control and *PIP5K1A* KD RKO cells. U6 snRNA was used as an endogenous control. All experiments were performed with at least three biological replicates. All data are represented as mean ± SEM. *P < 0.05, **P < 0.01, ***P < 0.001 and NS: not significant.

As PPK-1 is an ortholog of human PIP5K1A, we determined whether PIP5K1A regulates miRNA levels similar to *C. elegans* PPK-1. Firstly, we generated *PIP5K1A* stable knockdown (KD) in RKO colon cancer cells, a well-established model in miRNA biogenesis research involving pre-miRNA/XPO5 (37), by a lentivirus-based inducible shRNA system and confirmed knockdown of *PIP5K1A* by qRT-PCR (Figure 3A). Next, we investigated whether PIP5K1A regulates *let-7* miRNA expression. We found that knockdown of *PIP5K1A* showed increased mature *let-7a* and unchanged pre-*let-7a-1* levels compared to control cells by northern blotting and qRT-PCR assays (Figure 3B–E). We also found that mature *let-7b* and *let-7c* levels increased in *PIP5K1A* KD cells compared to control cells using a qRT-PCR assay (Figure 3E). Furthermore, *PIP5K1A* KD cells showed decreased pri-*let-7a-1* and pri-*let-7b* levels compared to control cells (Figure 3F). As pri-*let-7c* expression levels are very low in these cells, we did not investigate them.

We also tested the effect of overexpression (OE) of *PIP5K1A* on *let-7* levels by northern blotting and qRT-PCR experiments. Firstly, we confirmed that *PIP5K1A* mRNA expression levels increased in *PIP5K1A* OE cells compared to control cells (Figure 3G). We found that *PIP5K1A* OE cells showed decreased mature *let-7a* and unaltered pre-*let-7a-1* levels relative to wild type cells (Figure 3H-K). Mature *let-7b and let-7c* levels also decreased, as tested by qRT-PCR (Figure 3K). We also found that *PIP5K1A* OE cells showed increased pri-*let-7a-1* and pri-*let-7b* levels (Figure 3L). Given that the change in levels of pri-miRNA and mature miRNA are not concordant, these results indicate that PIP5K1A regulates *let-7* miRNA expression at least partially at the post-transcriptional level.

As PIP5K1A negatively regulates mature *let-7* levels, we tested whether the knockdown of *PIP5K1A* effects expression of downstream targets of *let-7* miRNAs, such as *LIN28A*, *MYC*, *HMGA2* (38). We found that those mRNA levels decreased in *PIP5K1A* KD compared to with control cells (Figure 3M). Furthermore, other miRNAs such as miR-122, miR-125a/miR-125b (orthologs of *C. elegans lin-4* miRNA (39)) and miR-886 levels increased in *PIP5K1A* KD cells, however some not significantly (Figure 3N). Taken together, these findings indicate that PIP5K1A negatively regulates mature miRNA levels similar to its *C. elegans* ortholog, PPK-1.

### PIP5K1A physically interacts with and co-localizes with XPO5

From our results above, PIP5K1A seemed to participate in regulating miRNA processing or biogenesis. Mammalian XPO5 belongs to the importin-beta family, a Ran-GTP-dependent dsRNA-binding protein that regulates the nuclear export of pre-miRNAs (6,40) and has a rate-limiting role in miRNA biogenesis. This led us to test whether there is a connection between PIP5K1A and XPO5. We confirmed that siRNA knockdown of *XPO5* indeed results in decreased mature *let-7a*, *let-7b* and *let-7c* levels compared to control cells using a qRT-PCR assay as previously reported (37) (Supplementary Figure S4).

**Figure 4.**
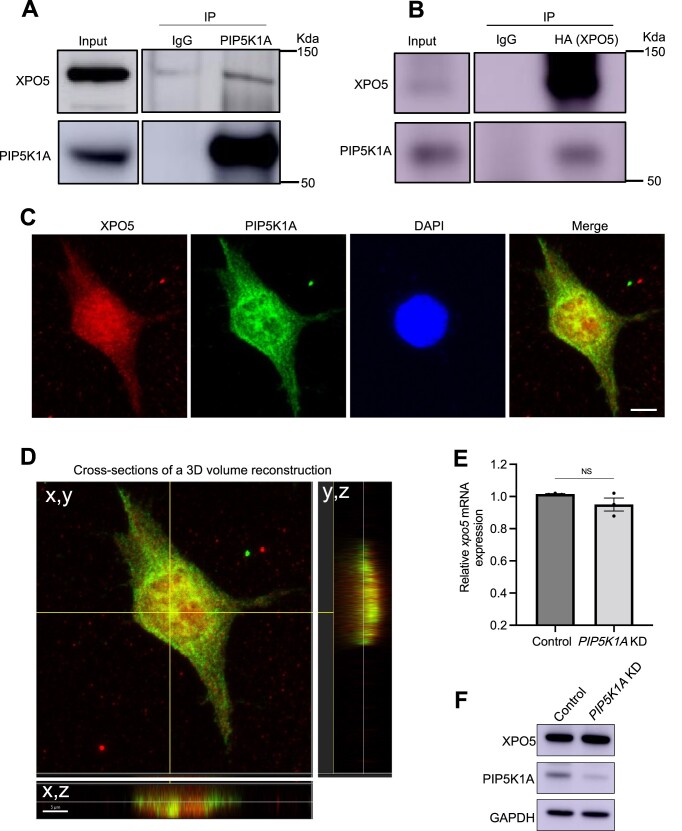
PIP5K1A physically interacts with and localizes with XPO5. (A, B) Physical interaction between PIP5K1A and XPO5. (**A**) Endogenous PIP5K1A of HEK293 cells was immunoprecipitated and then the XPO5 protein was analyzed by immunoblotting. Normal IgG was used as a negative control. (**B**) HEK293 cells were transfected with HA::XPO5 and XPO5 was immunoprecipitated with anti-HA antibody. Normal IgG was used as a negative. (**C**) Representative image of confocal imaging of co-localization between PIP5K1A and XPO5 in RKO cells. DAPI was used to stain the nuclear DNA. *n* = 30 cells, Scale bar: 7 μm. (**D**) Cross-sections of a 3D volume reconstruction. The presence of the colocalization signal was examined by generation of a 3D cross-section reconstructed from a confocal z-stack. The colocalization (yellow, arrow) of PIP5K1A (green) and XPO5 (red) in the nucleus in three dimensions (3D), as shown in the x,y, x,z, and y,z planes. (**E**) qRT-PCR analysis of *XPO5* mRNA levels in control and *PIP5K1A* KD RKO cells. *GAPDH* mRNA was used as an endogenous control. The data are represented as mean ± SEM. Individual experiments were performed in triplicate. NS: Not significant. (**F**) Western blot of XPO5 protein levels in control and *PIP5K1A* KD RKO cells. *GAPDH* mRNA was used as a loading control.

Firstly, we tested the simple hypothesis that PIP5K1A and XPO5 have a physical interaction. We immunoprecipitated PIP5K1A from HEK293 cell lysates with anti-PIP5K1A. We found that XPO5 co-precipitated with PIP5K1A (Figure 4A). However, immunoprecipitation of XPO5 with an anti-XPO5 antibody, did not show PIP5K1A co-precipitating with XPO5 (data not shown). As this anti-XPO5 antibody only recognizes the C-terminus of XPO5 (see Materials and Methods), we created an N-terminus tagged HA-XPO5 and performed the same experiments. We found that PIP5K1A co-precipitated with XPO5 when immunoprecipitation of XPO5 was with an anti-HA antibody (Figure 4B). We also used HA-KAP1(KRAB-associated protein) as a negative control to test the special interaction between PIP5K1A and XPO5. When we used HA antibody to immunoprecipitated KAP1, we did not detect PIP5K1A co-precipitating with KAP1 (Supplementary Figure S5). These results indicate that PIP5K1A physically interacts with XPO5 and seems to do so through its C-terminus.

To explore this interaction *in vivo*, the subcellular localization of the PIP5K1A-XPO5 complex was examined by immunofluorescence. We found that PIP5K1A co-localized with XPO5 mainly in the nucleus (Figure 4C). The presence of the colocalization signal within the nucleus was further examined by generation of a 3D cross-section reconstructed from a confocal z-stack, demonstrating that colocalization (yellow) of PIP5K1A (green) and XPO5 (red) occurs in the nucleus in three dimensions (3D), as shown in the x,y, x,z and y,z planes (Figure 4D). As the nuclear fraction of XPO5 binds with pre-miRNA in a Ran-GTP dependent manner and then exports the pre-miRNA to cytoplasm (6,7), this suggests that PIP5K1A may has a function with XPO5 in nucleus.

### PIP5K1A regulates the pre-miRNA/XPO5 complex during miRNA biogenesis

To address the question of how PIP5K1A functions in miRNA biogenesis, we tested whether PIP5K1A affects expression and/or function of XPO5. Because XPO5 is a known factor that has an important role in miRNA biogenesis and PIP5K1A associates with XPO5 from the above results, we tested the simple hypothesis that PIP5K1A regulates XPO5 levels and that accounts for its contribution to miRNA biogenesis. Thus, we analyzed the transcriptional and translational levels of XPO5 in *PIP5K1A* KD cells. We found that PIP5K1A does not affect *XPO5* mRNA and XPO5 protein levels (Figure 4E, F). This result indicates that PIP5K1A neither regulates XPO5 transcriptionally nor translationally.

**Figure 5.**
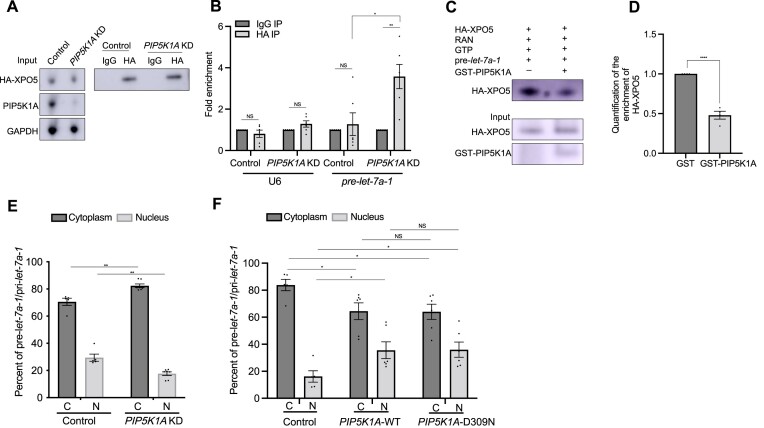
PIP5K1A regulates the binding ability of pre-miRNA/XPO5 complex. (A, B) RNA immunoprecipitation (RIP) assay. (**A**) RIP assay was employed by using anti-HA antibody and normal IgG antibody in control and *PIP5K1A* KD RKO cells. HA::XPO5 was transfected in control and *PIP5K1A* KD RKO cells and then HA::XPO5 was immunoprecipitated with anti-HA antibody. Normal IgG was used as a negative control. XPO5 protein was analyzed with anti-HA antibody by immunoblotting. GAPDH was used as a loading control. (**B**) Fold enrichment of *pre- let-7a-1* detected by qRT-PCR. U6 snRNA was used as a negative control. *n* = 6 independent biological replicates. (**C**) Western blot of XPO5 protein levels in 3′ biotin labeled pre-*let-7a-1* pull-down assay. Eluted HA-XPO5 protein was incubated with RAN, GTP and pre-*let-7a-1* in the absence or presence of the GST-PIP5K1A. HA-XPO5 and GST-PIP5K1A proteins were detected by western blotting with anti-HA and anti-GST antibodies, respectively. (**D**) Quantification of the enrichment of HA-XPO5. These results were normalized to the input of HA-XPO5 and calculated with ImageJ software. n = 3 independent biological replicates. Error bars represented as mean ± SEM. ****P < 0.0001. (**E, F**) The C/N (Cytoplasmic/Nuclear) ratio of pre-*let-7a-1/*pri-*let-7a-1* from the indicated cell lines by qRT-PCR analysis. All experiments were performed with at least three biological replicates. All data are represented as mean ± SEM. *P < 0.05, **P < 0.01, ****P < 0.0001 and NS: not significant.

As the C-terminal region of XPO5 is essential for the formation of the pre-miRNA/XPO5/Ran-GTP complex (37,41,42) and it is also important to interact with PIP5K1A from our results, this led us to hypothesize that PIP5K1A may have a role in the process of the formation of the pre-miRNA/XPO5 complex. For example, PIP5K1A binding may block pre-miRNA binding to the C-terminus of XPO5. If this is true, knockdown of PIP5K1A should increase the binding ability of pre-miRNA with XPO5. To test this hypothesis, we performed an RNA immunoprecipitation (RIP) assay in RKO cells. We expressed HA-XPO5 in *PIP5K1A* KD and control cells and immunoprecipitated XPO5 with the anti-HA antibody. We checked for XPO5 protein levels by western blot analysis (Figure 5A). Interestingly, we found that XPO5-associated pre-*let-7a-1* levels dramatically increased in cells with knockdown of *PIP5K1A* compared to control cells (Figure 5B). As a negative control, U6 snRNA levels were not changed (Figure 5B). Moreover, we used 3′ biotin labeled pre-*let-7a-1* and performed an RNA pull-down assay. We expressed HA-XPO5 in RKO cells and immunoprecipitated XPO5 with the anti-HA antibody. Eluted HA-XPO5 protein was incubated with RAN, GTP and 3′ biotin labeled pre-*let-7a-1* with or without GST-PIP5K1A. The experiment showed that pre-*let-7a-1*-associated HA-XPO5 levels were significantly reduced by addition of GST-PIP5K1A, compared to that without PIP5K1A (Figure 5C, D). We also confirmed the RNA pulldown efficiency and did not see a difference under these conditions (Supplementary Figure S6).

Furthermore, we also examined the cellular localization of XPO5 under depletion or overexpression of *PIP5K1A* by an immunofluorescence assay. We found a decreased C/N ratio of XPO5 localization in *PIP5K1A* OE compared to control cells (Supplementary Figure S7A–D) consistent with the idea that more PIP5K1A could bind with XPO5 to inhibit its function in the export of pre-miRNAs to cytoplasm. However, we did not observe a difference in the C/N ratio of XPO5 localization in *PIP5K1A* KD compared to control cells (Supplementary Figure S8A–C). Perhaps XPO5, after releasing its cargo in the cytoplasm, rapidly returns to the nucleus to mediate another round of transport.

These results indicate that PIP5K1A may function by blocking the binding of XPO5 to pre-*let-7a-1*. If this conclusion is correct, knockdown of *PIP5K1A* should increase pre-*let-7a-1* levels in the cytoplasmic fraction, because more pre-*let-7a-1* can bind with XPO5 and then be exported from the nucleus to the cytoplasm. So next we performed a qPCR assay to test this possibility. We note here that we used pre-*let-7a-1/*pri-*let-7a-1* levels to present the real pre-*let-7a-1* levels as a qPCR assay could not distinguish between pre-*let-7a-1* and pri-*let-7a-1*. As expected, we found knockdown of *PIP5K1A* resulted in higher pre-*let-7a-1/*pri-*let-7a-1* levels in cytoplasmic fraction compared to that in control cells (Figure 5E). We also found *PIP5K1A* OE resulted in lower pre-*let-7a-1*/pri-*let-7a-1* levels in the cytoplasmic fraction (Figure 5F), consistent with the decreased C/N ratio of XPO5 localization by immunofluorescent assay (Supplementary Figure S7D). However, U6 snRNA levels (a negative control) were not changed (Supplementary Figure S9). We confirmed the similar purity of cytoplasmic and nuclear fractions under each condition (Supplementary Figure S9). Taken together, these results suggest that PIP5K1A blocks the binding of pre-*let-7a-1* with XPO5 and then affects the C/N ratio of pre-miRNAs.

Next, we investigated whether the kinase activity of PIP5K1A is required for its function in miRNA biogenesis. First, we created a kinase-dead PIP5K1A mutant protein (*PIP5K1A*-D309N), which is already known to prevent the production of PIP2 (43). We confirmed this by immunofluorescence assay with an anti-PIP2 antibody (Supplementary Figure S10). We found that *PIP5K1A*-D309N OE cells showed decreased mature *let-7a and* unchanged pre-*let-7a-1* levels relative to wild type cells by northern blotting and qRT-PCR assays (Figure 3H–K), decreased mature *let-7b* and *let-7c* levels relative to wild type cells by qRT-PCR (Figure 3K) and lower pre-*let-7a-1*/pri-*let-7a-1* levels in the cytoplasmic fraction (Figure 5F) as also seen in *PIP5K1A*-WT OE cells. However, the pri-*let-7a-1* levels were not changed (Supplementary Figure S11). These results suggest that the kinase activity of PIP5K1A may be important for pri-*let-7* levels at an early step of miRNA biogenesis, however it is not required for regulating the export of pre-*let-7* from the nucleus and mature *let-7* levels at the later step of miRNA biogenesis.

## DISCUSSION

**Figure 6.**
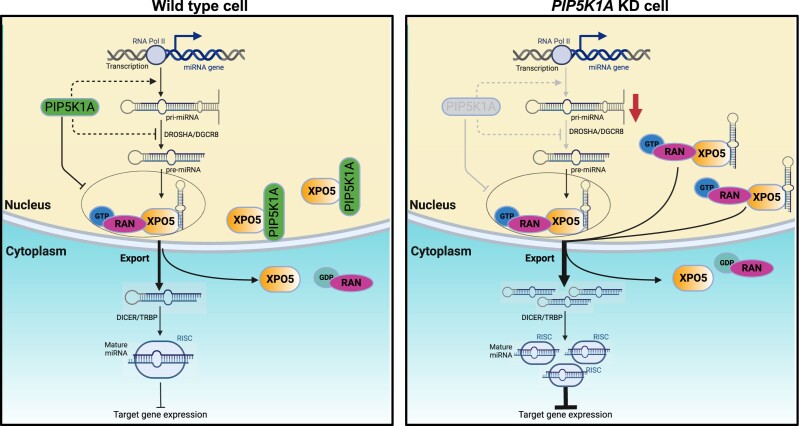
A schematic model of how PIP5K1A/XPO5 regulates pre-miRNA export from the nucleus to the cytoplasm.

Taken together, our study points to a previously unrecognized function of PIP5K1A in miRNA biogenesis. In summary, we have found that PIP5K1A regulates the ability of the pre-miRNA/XPO5 complex to participate in miRNA biogenesis/export. We propose a model based on our findings in RKO cell lines (Figure 6). In wild type cells, PIP5K1A interferes with the interaction between pre-miRNAs and XPO5 to maintain normal mature miRNA levels. Our model explains how PIP5K1A’s interference, which is reduced in *PIP5K1A* KD cells, allows more pre-miRNAs to bind to XPO5 and be exported to cytoplasm leading to more mature miRNAs to repress downstream gene expression.

Our study has demonstrated phosphatidylinositol-4-phosphate 5-kinase PPK-1/PIP5K1A regulates miRNA levels from *C. elegans* to humans. We have found that *ppk-1* mRNA directly binds to LIN-28 RNA protein from our LIN-28 CLIP-seq analysis (30) and that *ppk-1* mRNA levels decrease in a *lin-28*(*n719*) mutant compared to wild type animals by a qRT-PCR assay. Interestingly, we also found that in *ppk-1* RNAi animals, *lin-28* mRNA levels decreased compared to control RNAi animals. These results indicate that LIN-28 and PPK-1 function on each other in a feed forward loop. How this occurs will require further analysis. Consistent with *lin-28* and *ppk-1* acting positively on each other, we found that *ppk-1(lf)* leads to similar heterochronic phenotypes as *lin-28(lf). lin-28* mutant animals exhibit a decreased seam cell number as a result of skipping a symmetric cell division step in the L2 stage and exhibit precocious expression of adult cell fates. Similar to this, we also found a decreased seam cell number in *ppk-1* RNAi animals and a precocious adult cell fate phenotype. Loss of both genes also results in increased *let-7* levels.

To answer why *ppk-1* RNAi animals showed decreased *lin-28* mRNA levels, we speculate that it is possibly because the increase of mature *let-7* levels allows for increased negative regulation of its target *lin-28* mRNA. It has been reported that the human *PIP5K1A* mRNA is also bound by LIN28 (30,44) and PIP5K1A positively regulates *LIN28* mRNA levels from our results (Figure 3M), suggesting that it has a conserved mechanism from *C. elegans* to humans.

We found that PIP5K1A associates with the importin β-family factor XPO5 in human cells. While *C. elegans* PPK-1 is an ortholog of human PIP5K1A, unfortunately *C. elegans* lacks an XPO5 orthologue (45). However *C. elegans* has XPO-1, which is also an importin β-family and seems to function as the major nuclear export receptor (45). Our preliminary data indicate that PPK-1 associates with XPO-1 by a co-immunoprecipitation assay (unpublished data), however this possible interaction needs to be fully investigated in the future.

Our results point to further possibilities that warrant future investigation. For example, PIP5K1A may also have roles in other miRNA biogenesis steps, including (1) possibly transcriptional regulation (altered pri-miRNA levels), and (2) possibly Drosha/DGCR8 complex-mediated miRNA processing (unchanged pre-miRNA levels). We have found that pri*-let-7a-1* levels decreased in *PIP5K1A* KD cells, while its levels increased in *PIP5K1A-*WT OE cells, however there was no change in the *PIP5K1A*-D309N OE cells (Supplementary Figure S11). These results suggest that PIP5K1A positively regulates the pri-*let-7a-1* levels in a kinase dependent manner. As PIP5K1A kinase activity is reflected in PIP2 levels (confirmed through overexpression of *PIP5K1A*-WT OE or *PIP5K1A*-*D309N* OE as shown in Sup Fig. S10), we speculate that PIP5K1A regulates pri-miRNA levels through PIP2 levels. For example, PIP2 associates with histone H1 and RNA polymerase II in the nucleus. This possibility should be investigated in the future.

We have found that the pre-*let-7a-1* levels did not change upon both depletion or overexpression of PIP5K1A by northern blotting. Based on the results that PIP5K1A positively regulates pri-*let-7a-1* levels, we speculate that PIP5K1A may negatively regulate the Drosha/DGCR8 complex-mediated miRNA processing to keep normal pre-miRNA levels, but how this might happen remains unknown. As LIN28 co-transcriptionally binds pri-*let-7* and blocks its processing by Drosha/DGCR8 complex (35), they may function together in this step. It has been reported that XPO5 promotes primary miRNA processing in a RanGTP-independent manner (46) and XPO5 also interacts with Dicer mRNA to regulate Dicer expression post-transcriptionally (47). How these additional miRNA biogenesis steps are regulated by PIP5K1A/XPO5 should be investigated in the future.

A reason for why phosphatidylinositol 4-phosphate 5-kinase participates in miRNA biogenesis is not obvious, although it may be a response to signaling during cell cycle progression or for nuclear membrane integrity (48). It has been reported that rapid induction of XPO5 occurs during cell cycle entry by a PI3K-dependent post-transcriptional mechanism and inhibition of XPO5 results in a proliferation defect associated with a delayed G1/S transition. Tianyan *et al* also reported that PIP5K1A interacts with the cell cycle key regulator, CDK1 through formation of protein-complexes and regulates cell growth and survival (49). The localization of PIP5K1A and its product PIP2 in the nucleus has been reported by other groups and is confirmed by our results. The nuclear envelope (NE) is a physical barrier that regulates nucleocytoplasmic traffic and controls nuclear events. Since the maintenance of nuclear membrane integrity is essential for normal cell function, we speculate that it may make sense to connect nuclear PIP5K1A with miRNA biogenesis, however this should be addressed in the future. Given that miRNAs are implicated in multiple diseases (50), including cancer, this new finding might provide a novel therapeutic opportunity to modulate the levels of disease-associated miRNAs.

## Supplementary Material

gkad709_Supplemental_Files

## Data Availability

The datasets generated during and/or analyzed during the current study are available from the corresponding author upon request.

## Notes

Present address: Bohyung Yoon, i-SENS, Inc. 43 Banpo-daero 28-gil, Seocho-gu, Seoul 06646, South Korea.

Present address: Giovanni Stefani, Royal Brompton Hospital, Sydney Street, London SW3 6NP, UK.
