# Supplementary material for: Lipid kinase PIP5K1A regulates let-7 microRNA biogenesis through interacting with nuclear export protein XPO5
Source: Nucleic Acids Res. 2023 Sep 1;51(18):9849–62. doi: 10.1093/nar/gkad709 (PMC10570020; doi:10.1093/nar/gkad709)
Supplement: gkad709_Supplemental_Files [file gkad709_supplemental_files.zip › Li- sup figure .pdf]

Figure S1.

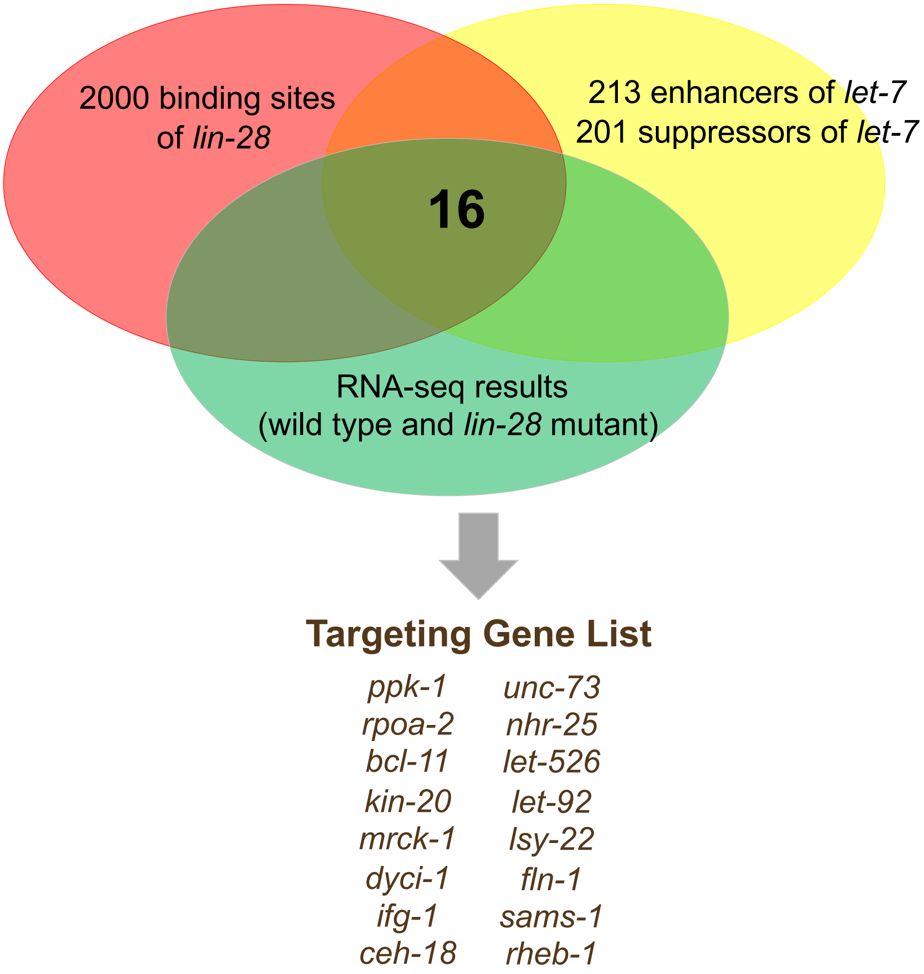

Figure S1. Experimental strategy. 16 candidate genes are listed, which were obtained from the overlap of these groups.

Figure S2.

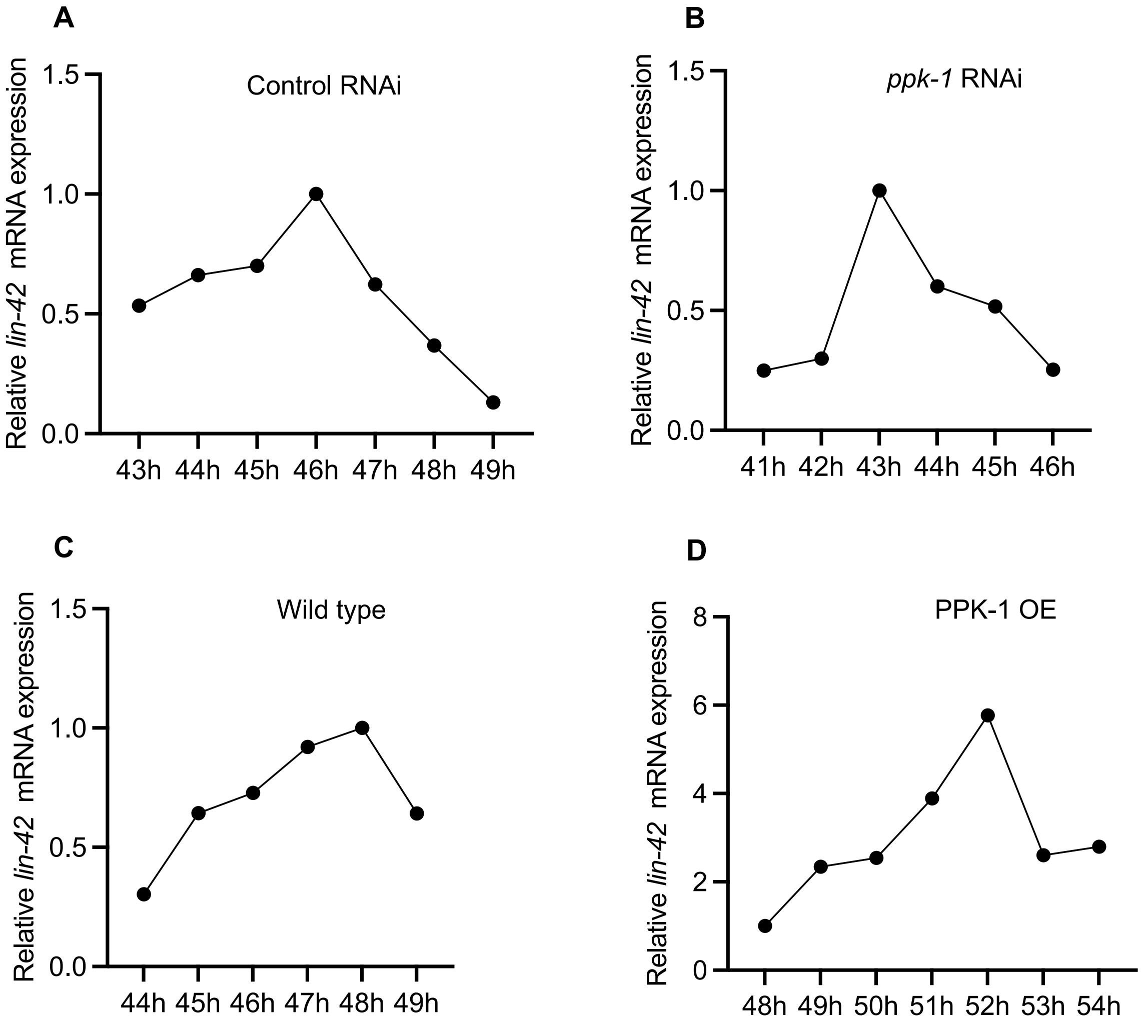

Figure S2. Time course of *lin-42* expression. qRT-PCR analysis of *lin-42* mRNA levels at different time points during the L4 larval stage in control RNAi (**A**), *ppk-1*(RNAi) (**B**), wild type (**C**) and PPK-1 OE (**D**) animals. *act-1* mRNA was used as an endogenous control. All experiments were performed at 20°C.

Figure S3.

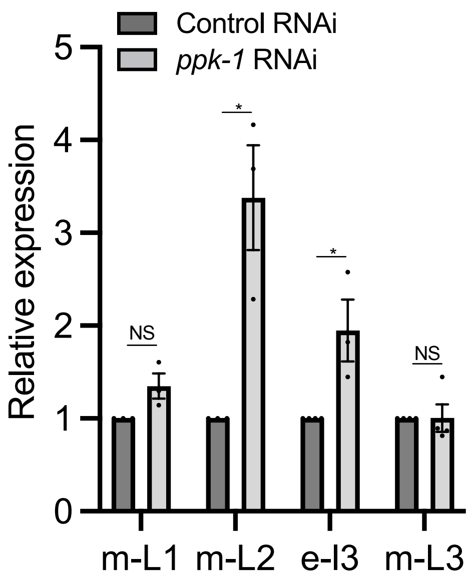

Figure S3. qRT-PCR analysis of mature *let-7* levels in control RNAi and *ppk-1*(RNAi) animals at the indicated stage. U18 snoRNA was used as an endogenous control. m-L1, mid-L1; m-L2, mid-L2; e-L3, early L3; m-L3, mid-L3. All experiments were performed with three biological replicates. All data are represented as mean ± SEM. *$p < 0.05$ and NS: Not significant.

Figure S4.

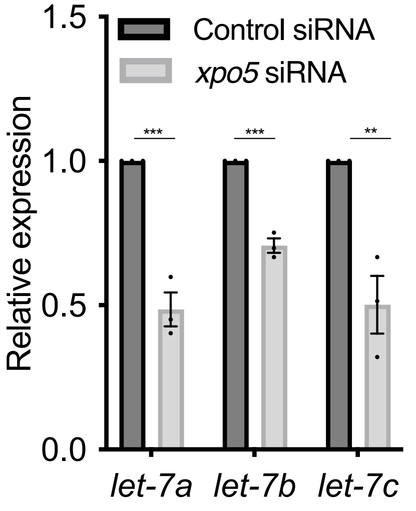

Figure S4. Depletion of *XPO5* results in decreased mature *let-7* expression. qRT-PCR analysis of mature *let-7a*, *let-7b* and *let-7c* levels in control and *xpo5* siRNA RKO cells. Represented as mean ± SEM. U6 snRNA was used as an endogenous control. Individual experiments were performed in triplicate. $^{**}p < 0.01$, $^{***}p < 0.001$.

Figure S5.

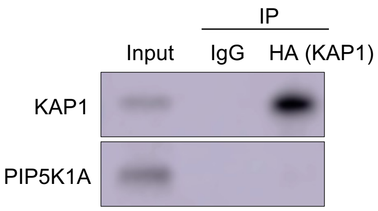

Figure S5. Co-immunoprecipitation assays. HEK293 cells were transfected with HA::KAP1 and KAP1 was immunoprecipitated with anti-HA antibody then the PIP5K1A protein was analyzed by immunoblotting. Normal IgG was used as a negative.

Figure S6.

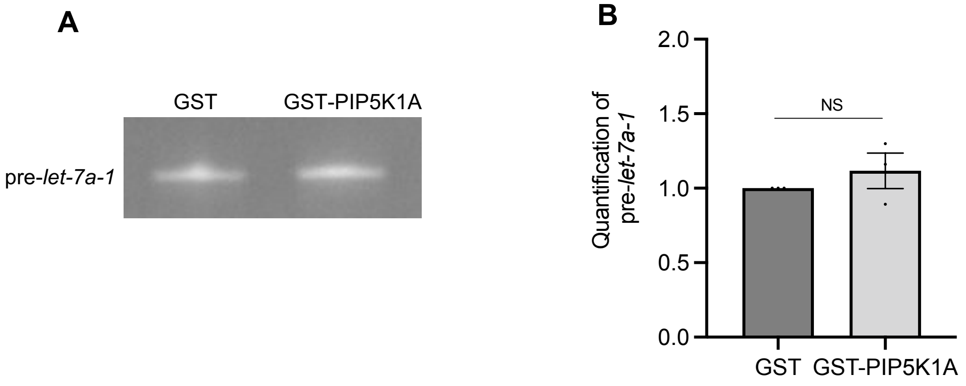

Figure S6. RNA pulldown efficiency of the pre-*let-7a-1*. **(A)** pre-*let-7a-1* levels in the RNA pulldown assay under with PIP5K1A or without. **(B)** Quantifications of pre-*let-7a-1* levels. n=3 independent biological replicates.

Figure S7.

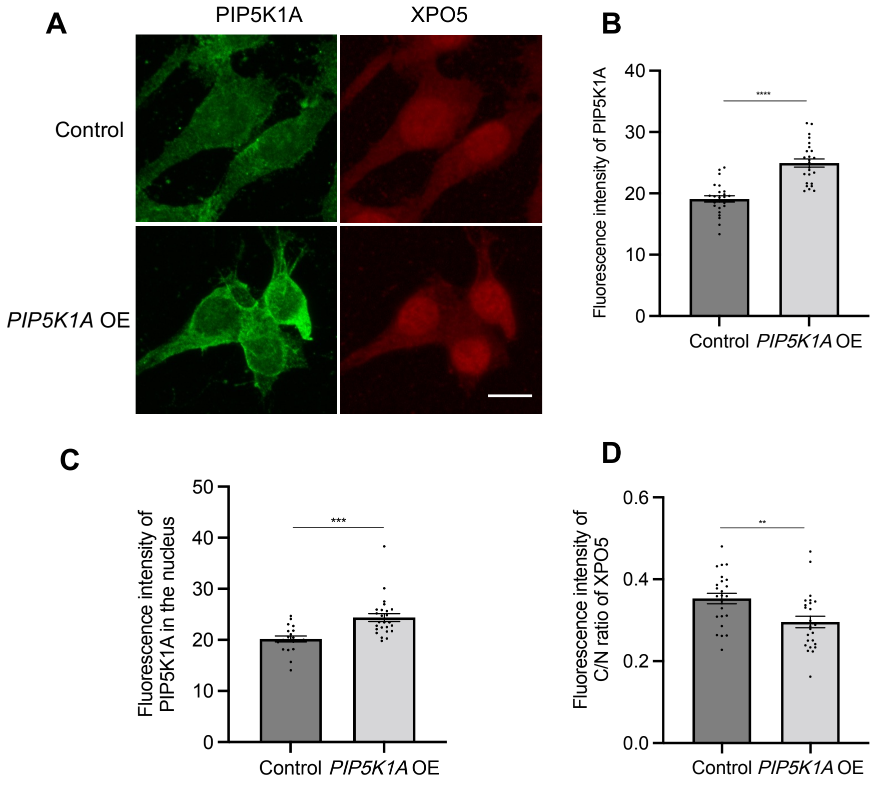

Figure S7. Cellular localization of XPO5 in control and *PIP5K1A* OE RKO cells by immunofluorescence assay. **(A)** Confocal imaging of XPO-5 (red) and PIP5K1A (green). Quantifications of the fluorescence intensity of whole PIP5K1A **(B)**, nuclear PIP5K1A **(C)** and the C/N ratio of XPO5 localization **(D)** were performed by Image J. n>20 cells. All data are represented as mean ± SEM. Scale bar: 10μm. ****p < 0.0001 and **p < 0.01.

Figure S8.

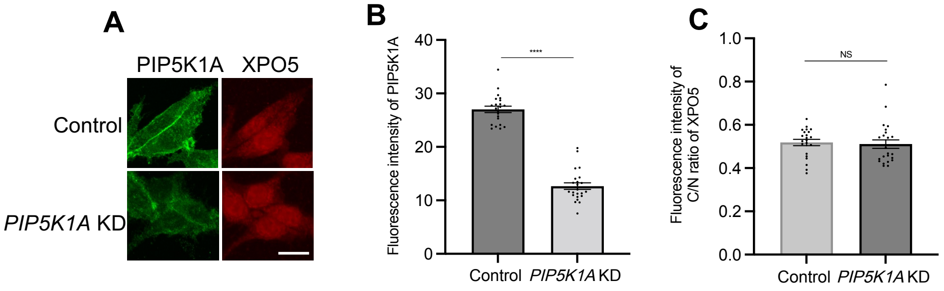

Figure S8. Cellular localization of XPO5 in control and *PIP5K1A* KD RKO cells by immunofluorescence assay. **(A)** Confocal imaging of XPO-5 (red) and PIP5K1A (green). Quantifications of the fluorescence intensity of PIP5K1A **(B)** and the C/N ratio of XPO5 localization **(C)** were performed by Image J. n>20 cells. All data are represented as mean ± SEM. Scale bar: 10μm. ****$p < 0.0001$ and NS: Not significant.

Figure S9.

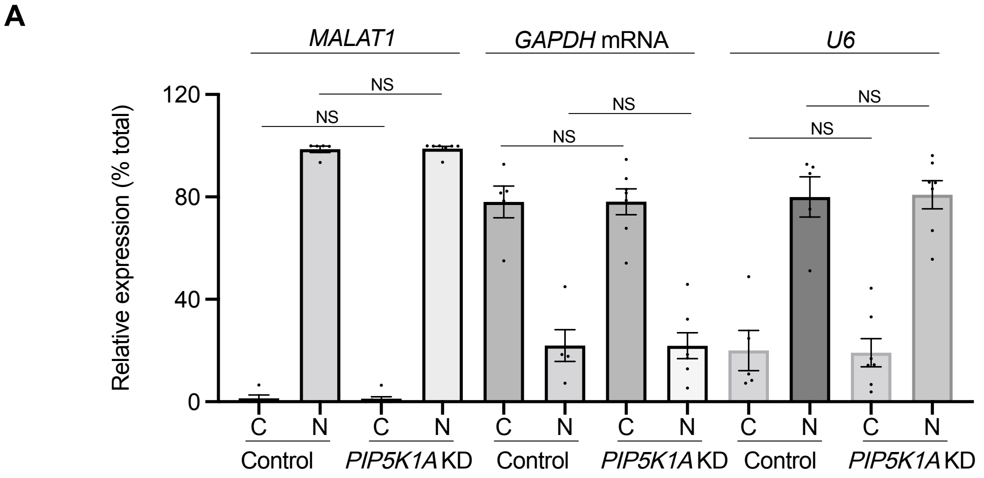

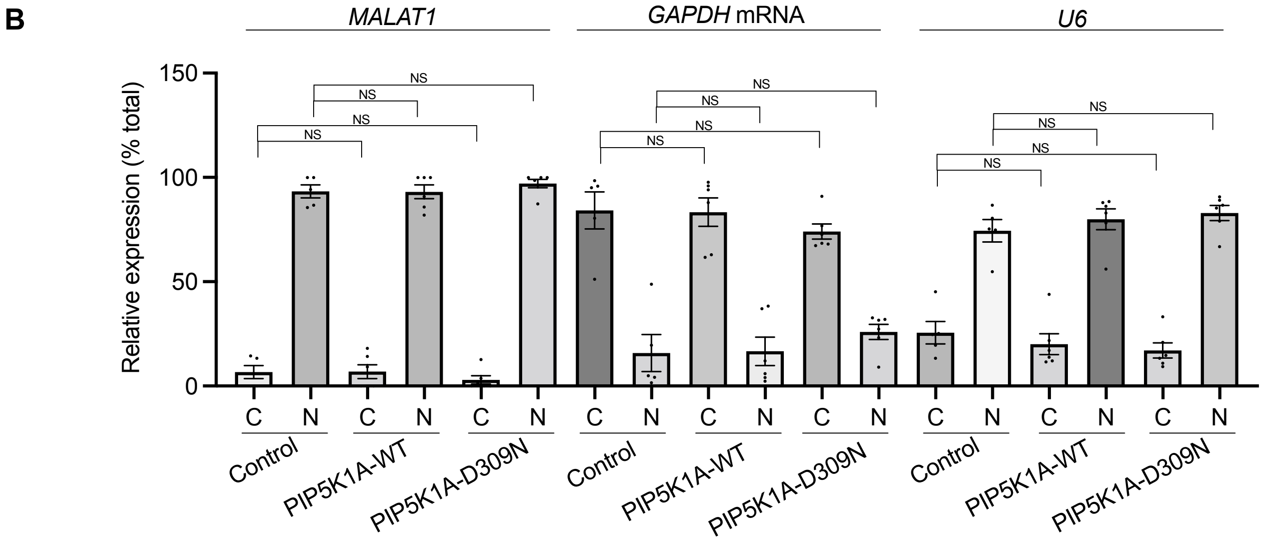

Figure S9. The C/N (Cytoplasmic/Nuclear) ratio of *MALAT1*, *GAPDH* mRNA and U6 snRNA from the indicated cell lines by qRT-PCR analysis **(A, B)**. *MALAT1* and *GAPDH* mRNA as a nuclear and cytoplasmic fractions marker, respectively. U6 snRNA was used as a negative control. All data are represented as mean ± SEM. **p* < 0.05 and NS: Not significant.

Figure S10.

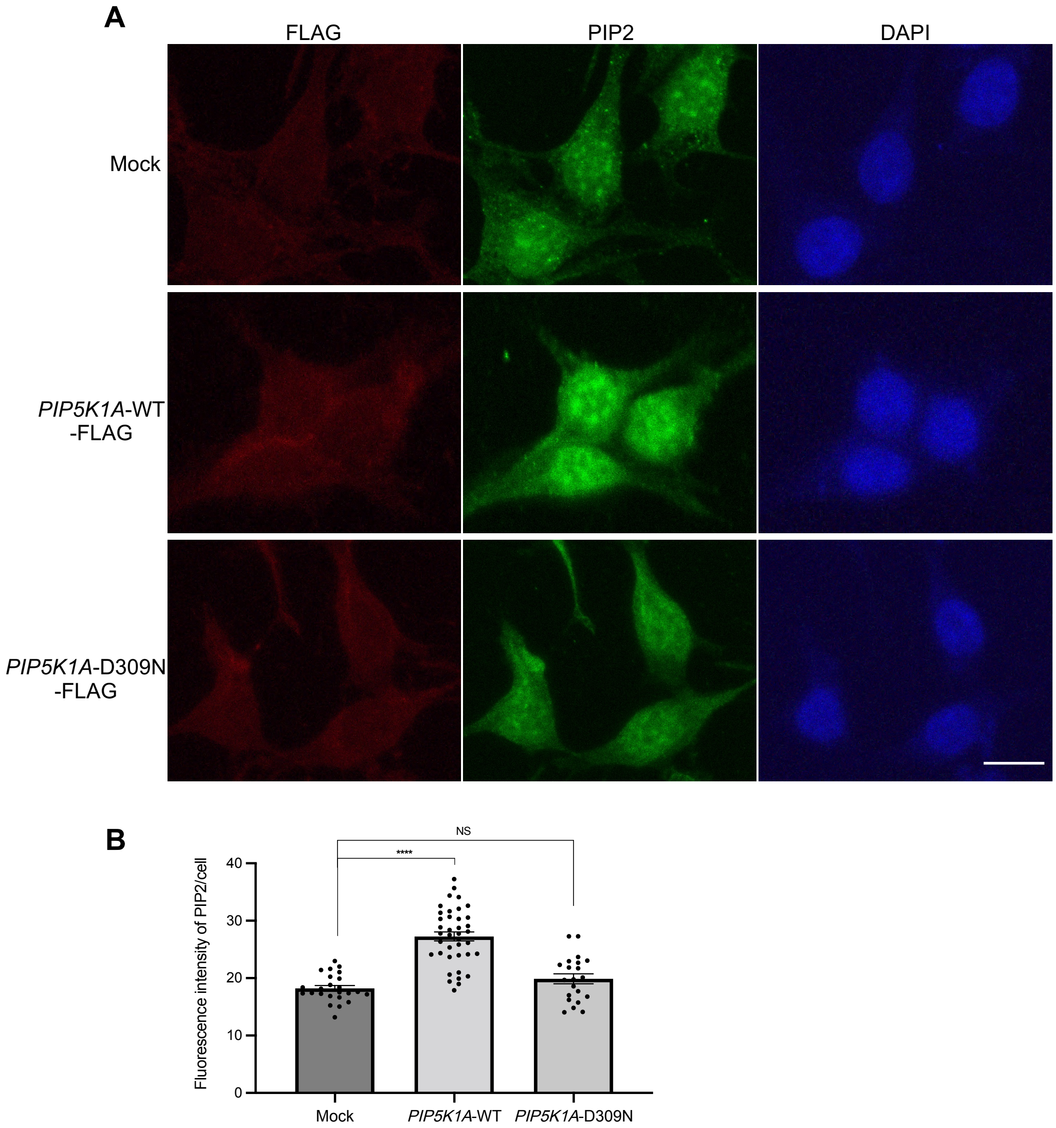

Figure S10. *PIP5K1A*-D309N mutant abolishes the kinase activity. **(A)** RKO cells were transfected with mock and a FLAG-tagged *PIP5K1A*-WT or a kinase dead *PIP5K1A*-D309N mutant gene. PIP2 levels were visualized by a PIP2 antibody. Scale bar: 10μm. **(B)** Fluorescence intensity of PIP2 was determined by ImageJ**.** ****p < 0.0001 and NS: Not significant. n > 20, All data are represented as mean ± SEM.

Figure S11.

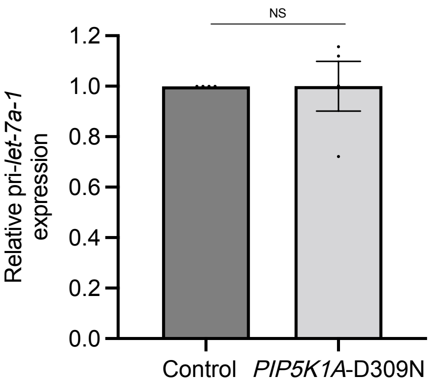

Figure S11. qRT-PCR analysis of pri-*let-7a-1* levels from control and *PIP5K1A*-D307N OE cells. *GAPDH* mRNA was used as an endogenous control. The experiment was performed with three biological replicates. The data are represented as mean ± SEM. NS: Not significant.
