# Supplementary material for: Lipid kinase PIP5K1A regulates let-7 microRNA biogenesis through interacting with nuclear export protein XPO5
Source: Nucleic Acids Res. 2023 Sep 1;51(18):9849–62. doi: 10.1093/nar/gkad709 (PMC10570020; doi:10.1093/nar/gkad709)
Supplement: gkad709_Supplemental_Files [file gkad709_supplemental_files.zip › Supplemental Table 1 legends.docx]

**Supplemental Table 1**

RNA-seq analysis between wild type and *lin-28* (*n719*) animals at the late L1 stage. RNA-seq was described in reference (30). Differential expression testing was performed using DESeq2 by the HSPH Bioinformatics Core.
